# Updates in Cardiology

**DOI:** 10.1590/S1679-45082013000300001

**Published:** 2013

**Authors:** Miguel Cendoroglo

**Affiliations:** 1 Hospital Israelita Albert Einstein, São Paulo, SP, Brazil.

In this issue of **einstein**, Cardiology is the central theme. Dr. Marcia Makdisse and Dr. Marcelo Katz, our guest-editors, joined our editorial board on the efforts in bringing this issue to publication.

As a result, scientific relevant articles, published in this issue, represent the growing importance of **einstein**. Focusing on quality of the published articles, **einstein** reached PubMed indexing, which endorses the journal as a place for credible scientific publishing - proof of this is the range of institutions that are present in this edition. In addition, papers also represent the scientific evolution of Cardiology at *Hospital Israelita Albert Einstein*.

Initially, I emphasize the diversity of articles on this issue that will connect readers with a wide variety of topics in Cardiology.

Approaches on themes, such as decompensated heart failure and vascular calcification will enable the reader to catch up on issues present in daily clinical practice, as well as knowing the clinical implications of these phenomena.

To illustrate how new technologies can be incorporated, improving the practice of Cardiology, highlighted topics are 320-row multidetector computed tomography coronary angiography, contrast echocardiography in myocardial infarction and robotic cardiac surgery.

Other articles also address essential themes, such as: adherence to pharmacological and non-pharmacological treatment, systemic hypertension, percutaneous coronary intervention, pulmonary thromboembolism, obesity, metabolic syndrome, biomarkers, cardiac rehabilitation and Chaga's disease, guidelines for the use of blood components and congenital heart disease.

Interesting insights come from the approach on hyperbaric oxygen therapy as adjuvant treatment for mediastinitis after coronary artery bypass graft surgery.

Case reports arouse the reader's attention, for their originality and relevance. Finally, on the Health Economics and Management section, an institutional guideline on myocardial infarction is presented, as well as the steps for its implementation, the challenges and measures to achieve compliance with quality indicators and monitoring clinical outcomes.

I believe that this thematic issue will contribute to the quality of scientific diffusion of knowledge. The reader will be updated on current and relevant issues. Enjoy your reading!

